# Supplementary material for: Trends and disparities in dilated cardiomyopathy related mortality among adults in the United States: A CDC WONDER analysis (1999–2023)
Source: PLoS One. 2025 Oct 16;20(10):e0333525. doi: 10.1371/journal.pone.0333525 (PMC12530569; doi:10.1371/journal.pone.0333525)
Supplement: S1 Table — (DOCX) [file pone.0333525.s001.docx]

**Supplemental Table 1: Dilated Cardiomyopathy related deaths, stratified by Sex and Race in the United States, 1999 to 2023.**

|  | Deaths | | | | | |
| --- | --- | --- | --- | --- | --- | --- |
| Year | **Female** | **Male** | **NH White** | **NH Black or African American** | **NH other** | **Hispanic or Latino** |
| 1999 | 3324 | 5863 | 6547 | 1900 | 204 | 495 |
| 2000 | 3072 | 5617 | 6079 | 1907 | 200 | 466 |
| 2001 | 2900 | 5426 | 5860 | 1813 | 200 | 434 |
| 2002 | 2793 | 5321 | 5738 | 1678 | 218 | 435 |
| 2003 | 2674 | 5034 | 5441 | 1641 | 183 | 429 |
| 2004 | 3884 | 6219 | 7436 | 1858 | 265 | 512 |
| 2005 | 3630 | 6064 | 7088 | 1812 | 246 | 526 |
| 2006 | 3317 | 5572 | 6500 | 1669 | 200 | 494 |
| 2007 | 3131 | 5259 | 6137 | 1566 | 207 | 466 |
| 2008 | 3049 | 5200 | 6004 | 1529 | 205 | 491 |
| 2009 | 2812 | 4980 | 5646 | 1428 | 220 | 473 |
| 2010 | 2652 | 4751 | 5352 | 1365 | 172 | 493 |
| 2011 | 2493 | 4505 | 5059 | 1232 | 212 | 476 |
| 2012 | 2376 | 4287 | 4841 | 1153 | 204 | 446 |
| 2013 | 2316 | 4221 | 4750 | 1098 | 219 | 454 |
| 2014 | 2116 | 3988 | 4347 | 1014 | 220 | 494 |
| 2015 | 2127 | 4010 | 4516 | 983 | 187 | 424 |
| 2016 | 2119 | 3892 | 4285 | 1040 | 221 | 446 |
| 2017 | 2119 | 3949 | 4323 | 1004 | 232 | 483 |
| 2018 | 2017 | 3799 | 4046 | 1072 | 247 | 435 |
| 2019 | 1988 | 3837 | 4051 | 1039 | 201 | 512 |
| 2020 | 2143 | 4304 | 4341 | 1246 | 247 | 598 |
| 2021 | 2187 | 4372 | 4543 | 1111 | 244 | 579 |
| 2022 | 2119 | 4345 | 4397 | 1167 | 227 | 573 |
| 2023 | 1917 | 3983 | 4031 | 1043 | 202 | 543 |

**NH,** non-Hispanic.
