# Supplementary material for: Trends and disparities in dilated cardiomyopathy related mortality among adults in the United States: A CDC WONDER analysis (1999–2023)
Source: PLoS One. 2025 Oct 16;20(10):e0333525. doi: 10.1371/journal.pone.0333525 (PMC12530569; doi:10.1371/journal.pone.0333525)
Supplement: S2 Table — (DOCX) [file pone.0333525.s002.docx]

**Supplemental Table 2: Annual percent change (APC) of Dilated Cardiomyopathy age-adjusted mortality rates per 100,000 in the United States, 1999 to 2023**

| **Year Interval** | **APC (95% CI)** |
| --- | --- |
| **Overall** |  |
| 1999-2002 | -6.197 (-12.4204 - -0.718) |
| 2002-2005 | 5.0694 (-6.7512 - 8.1592) |
| 2005-2014 | -6.8387 (-9.0009 - -3.0529) |
| 2014-2023 | -0.8104 (-2.0172 - 0.7092) |
| **Male** |  |
| 1999-2005 | -0.8299 (-3.2317 - 5.3831) |
| 2005-2016 | -5.8994 (-11.5339 - -4.8516) |
| 2016-2023 | 0.452 (-1.9612 - 5.6601) |
| **Female** |  |
| 1999-2002 | -7.2584 (-14.7913 - -2.0437) |
| 2002-2005 | 7.3739 (-0.4885 - 11.1428) |
| 2005-2014 | -7.1764 (-10.2727 - -5.9345) |
| 2014-2023 | -1.3674 (-2.672 - 0.4515) |
| **NH White** |  |
| 1999-2002 | -5.3444 (-12.1895 - 0.5497) |
| 2002-2005 | 6.5258 (-6.1174 - 9.9088) |
| 2005-2014 | -6.3111 (-9.136 - -2.9566) |
| 2014-2023 | -0.5172 (-2.2442 - 1.8081) |
| **NH Black or African American** |  |
| 1999-2005 | -2.7915 (-4.5525 - 0.4018) |
| 2005-2015 | -7.9056 (-10.8567 - -6.951) |
| 2015-2023 | 0.1344 (-1.6026 - 2.5754) |
| **NH other** |  |
| 1999-2023 | -4.3244 (-4.9796 - -3.6271) |
| **Hispanic or Latino** |  |
| 1999-2016 | -5.428 (-8.9097 - -4.4902) |
| 2016-2023 | -0.1059 (-3.4182 - 10.0454) |
| **Rural areas** |  |
| 1999-2004 | 1.7037 (-2.3964 - 14.5594) |
| 2004-2020 | -3.9935 (-6.8322 - -3.2044) |
| **Urban areas** |  |
| 1999-2002 | -6.5454 (-12.8257 - -2.1723) |
| 2002-2005 | 4.7056 (-1.6686 - 7.8661) |
| 2005-2014 | -6.9283 (-10.2407 - -5.8099) |
| 2014-2020 | -1.54 (-3.9383 - 3.6454) |
