# Supplementary material for: Trends and disparities in dilated cardiomyopathy related mortality among adults in the United States: A CDC WONDER analysis (1999–2023)
Source: PLoS One. 2025 Oct 16;20(10):e0333525. doi: 10.1371/journal.pone.0333525 (PMC12530569; doi:10.1371/journal.pone.0333525)
Supplement: S3 Table — (DOCX) [file pone.0333525.s003.docx]

**Supplemental Table 3: Overall and Sex‐Stratified** **Dilated Cardiomyopathy Age-Adjusted Mortality Rates per 100,000 in the United States, 1999 to 2023**

|  | **Age-Adjusted Rate (95% CI)** | | |
| --- | --- | --- | --- |
| **Year** | **Male** | **Female** | **Overall** |
| **1999** | 7.74 (7.54 - 7.94) | 3.26 (3.14 - 3.37) | 5.19 (5.08 - 5.29) |
| **2000** | 7.33 (7.13 - 7.52) | 2.96 (2.85 - 3.06) | 4.82 (4.72 - 4.93) |
| **2001** | 6.92 (6.73 - 7.11) | 2.75 (2.65 - 2.86) | 4.55 (4.46 - 4.65) |
| **2002** | 6.67 (6.49 - 6.85) | 2.65 (2.55 - 2.75) | 4.38 (4.28 - 4.47) |
| **2003** | 6.15 (5.98 - 6.32) | 2.48 (2.39 - 2.58) | 4.08 (3.99 - 4.17) |
| **2004** | 7.71 (7.51 - 7.9) | 3.48 (3.37 - 3.59) | 5.29 (5.19 - 5.39) |
| **2005** | 7.29 (7.1 - 7.47) | 3.15 (3.04 - 3.25) | 4.96 (4.86 - 5.05) |
| **2006** | 6.54 (6.36 - 6.71) | 2.88 (2.78 - 2.98) | 4.48 (4.39 - 4.58) |
| **2007** | 6.03 (5.87 - 6.2) | 2.67 (2.57 - 2.76) | 4.17 (4.08 - 4.26) |
| **2008** | 5.83 (5.67 - 6) | 2.56 (2.47 - 2.65) | 4.01 (3.93 - 4.1) |
| **2009** | 5.46 (5.3 - 5.61) | 2.36 (2.27 - 2.45) | 3.71 (3.62 - 3.79) |
| **2010** | 5.15 (5 - 5.29) | 2.16 (2.08 - 2.24) | 3.48 (3.4 - 3.56) |
| **2011** | 4.73 (4.59 - 4.87) | 1.98 (1.9 - 2.06) | 3.22 (3.15 - 3.3) |
| **2012** | 4.34 (4.21 - 4.47) | 1.88 (1.8 - 1.96) | 3.00 (2.93 - 3.07) |
| **2013** | 4.22 (4.09 - 4.35) | 1.82 (1.74 - 1.89) | 2.91 (2.84 - 2.98) |
| **2014** | 3.88 (3.76 - 4.01) | 1.63 (1.56 - 1.7) | 2.66 (2.59 - 2.72) |
| **2015** | 3.82 (3.7 - 3.94) | 1.62 (1.55 - 1.69) | 2.62 (2.55 - 2.68) |
| **2016** | 3.66 (3.54 - 3.77) | 1.62 (1.55 - 1.69) | 2.55 (2.49 - 2.62) |
| **2017** | 3.63 (3.51 - 3.74) | 1.59 (1.52 - 1.66) | 2.55 (2.48 - 2.61) |
| **2018** | 3.43 (3.31 - 3.54) | 1.49 (1.42 - 1.55) | 2.39 (2.33 - 2.46) |
| **2019** | 3.4 (3.29 - 3.51) | 1.43 (1.37 - 1.5) | 2.36 (2.30 - 2.42) |
| **2020** | 3.79 (3.67 - 3.9) | 1.51 (1.45 - 1.58) | 2.56 (2.50 - 2.62) |
| **2021** | 3.87 (3.75 - 3.98) | 1.61 (1.54 - 1.68) | 2.65 (2.59 - 2.72) |
| **2022** | 3.75 (3.64 - 3.87) | 1.48 (1.42 - 1.55) | 2.53 (2.47 - 2.60) |
| **2023** | 3.4 (3.29 - 3.51) | 1.38 (1.32 - 1.45) | 2.34 (2.28 - 2.40) |
