# Supplementary material for: Trends and disparities in dilated cardiomyopathy related mortality among adults in the United States: A CDC WONDER analysis (1999–2023)
Source: PLoS One. 2025 Oct 16;20(10):e0333525. doi: 10.1371/journal.pone.0333525 (PMC12530569; doi:10.1371/journal.pone.0333525)
Supplement: S4 Table — (DOCX) [file pone.0333525.s004.docx]

**Supplemental Table 4: Dilated Cardiomyopathy Related Age-Adjusted Mortality Rates per 100,000, Stratified by Race in the United States, 1999 to 2023**

|  | Age-Adjusted Rate (95% CI) | | | |
| --- | --- | --- | --- | --- |
| Year | **NH White** | **NH Black or African American** | **NH other** | **Hispanic or Latino** |
| 1999 | 4.51 (4.4 - 4.62) | 11.2 (10.69 - 11.72) | 3.73 (3.19 - 4.28) | 4.9 (4.44 - 5.37) |
| 2000 | 4.16 (4.05 - 4.26) | 11.18 (10.67 - 11.69) | 3.39 (2.89 - 3.89) | 4.21 (3.79 - 4.62) |
| 2001 | 3.95 (3.85 - 4.05) | 10.22 (9.74 - 10.7) | 3.12 (2.66 - 3.58) | 3.71 (3.34 - 4.09) |
| 2002 | 3.88 (3.78 - 3.98) | 9.28 (8.82 - 9.73) | 3.27 (2.81 - 3.73) | 3.48 (3.13 - 3.84) |
| 2003 | 3.62 (3.52 - 3.71) | 8.84 (8.4 - 9.27) | 2.71 (2.29 - 3.12) | 3.28 (2.94 - 3.62) |
| 2004 | 4.84 (4.73 - 4.95) | 10.05 (9.58 - 10.52) | 3.7 (3.23 - 4.18) | 3.93 (3.57 - 4.3) |
| 2005 | 4.59 (4.48 - 4.69) | 9.49 (9.04 - 9.94) | 3.19 (2.76 - 3.61) | 3.82 (3.47 - 4.18) |
| 2006 | 4.12 (4.02 - 4.22) | 8.51 (8.09 - 8.93) | 2.48 (2.12 - 2.84) | 3.27 (2.96 - 3.58) |
| 2007 | 3.88 (3.78 - 3.98) | 7.72 (7.32 - 8.11) | 2.55 (2.18 - 2.91) | 2.99 (2.7 - 3.29) |
| 2008 | 3.75 (3.65 - 3.84) | 7.38 (7 - 7.76) | 2.32 (1.99 - 2.66) | 3 (2.71 - 3.29) |
| 2009 | 3.51 (3.41 - 3.6) | 6.76 (6.4 - 7.12) | 2.33 (2.01 - 2.65) | 2.61 (2.35 - 2.86) |
| 2010 | 3.27 (3.18 - 3.36) | 6.42 (6.07 - 6.77) | 1.75 (1.48 - 2.02) | 2.73 (2.47 - 2.99) |
| 2011 | 3.04 (2.95 - 3.13) | 5.66 (5.34 - 5.99) | 1.99 (1.72 - 2.27) | 2.46 (2.23 - 2.7) |
| 2012 | 2.87 (2.79 - 2.95) | 5.05 (4.75 - 5.35) | 1.81 (1.55 - 2.07) | 2.09 (1.88 - 2.3) |
| 2013 | 2.82 (2.74 - 2.91) | 4.75 (4.46 - 5.04) | 1.87 (1.61 - 2.12) | 2.06 (1.86 - 2.26) |
| 2014 | 2.55 (2.47 - 2.63) | 4.28 (4.01 - 4.55) | 1.74 (1.5 - 1.97) | 2.16 (1.96 - 2.37) |
| 2015 | 2.63 (2.55 - 2.71) | 3.99 (3.74 - 4.25) | 1.42 (1.21 - 1.63) | 1.69 (1.52 - 1.86) |
| 2016 | 2.48 (2.41 - 2.56) | 4.14 (3.88 - 4.4) | 1.65 (1.43 - 1.87) | 1.72 (1.55 - 1.89) |
| 2017 | 2.51 (2.43 - 2.59) | 3.92 (3.67 - 4.17) | 1.56 (1.36 - 1.77) | 1.8 (1.63 - 1.97) |
| 2018 | 2.31 (2.23 - 2.38) | 4.1 (3.85 - 4.35) | 1.66 (1.45 - 1.87) | 1.55 (1.4 - 1.7) |
| 2019 | 2.29 (2.22 - 2.37) | 3.85 (3.61 - 4.09) | 1.31 (1.13 - 1.5) | 1.78 (1.62 - 1.94) |
| 2020 | 2.42 (2.35 - 2.5) | 4.51 (4.26 - 4.77) | 1.56 (1.36 - 1.76) | 2 (1.83 - 2.16) |
| 2021 | 2.68 (2.6 - 2.76) | 4.09 (3.84 - 4.34) | 1.58 (1.38 - 1.78) | 1.86 (1.7 - 2.02) |
| 2022 | 2.52 (2.44 - 2.6) | 4.27 (4.02 - 4.52) | 1.41 (1.23 - 1.6) | 1.79 (1.64 - 1.94) |
| 2023 | 2.3 (2.22 - 2.37) | 3.77 (3.53 - 4) | 1.23 (1.06 - 1.41) | 1.67 (1.53 - 1.82) |

NH = non-Hispanic.
