# Supplementary material for: Trends and disparities in dilated cardiomyopathy related mortality among adults in the United States: A CDC WONDER analysis (1999–2023)
Source: PLoS One. 2025 Oct 16;20(10):e0333525. doi: 10.1371/journal.pone.0333525 (PMC12530569; doi:10.1371/journal.pone.0333525)
Supplement: S5 Table — (DOCX) [file pone.0333525.s005.docx]

**Supplemental Table 5: Dilated Cardiomyopathy Related Age-Adjusted Mortality Rates per 100,000, Stratified by States in the United States, 1999 to 2023**

| **State** | **Age-Adjusted Rate (95%CI)** | |
| --- | --- | --- |
|  | **1999-2020** | **2021-2023** |
| **Alabama** | 2.64 (2.52 - 2.75) | 1.63 (1.38 - 1.87) |
| **Alaska** | 3.93 (3.44 - 4.41) | 3.26 (2.37 - 4.38) |
| **Arizona** | 2.83 (2.72 - 2.94) | 3.07 (2.79 - 3.34) |
| **Arkansas** | 2.33 (2.19 - 2.47) | 1.12 (0.88 - 1.4) |
| **California** | 4.17 (4.12 - 4.23) | 2.97 (2.85 - 3.09) |
| **Colorado** | 1.84 (1.74 - 1.95) | 2.08 (1.82 - 2.34) |
| **Connecticut** | 1.91 (1.8 - 2.02) | 0.89 (0.7 - 1.12) |
| **Delaware** | 5.55 (5.15 - 5.94) | 4.21 (3.41 - 5.01) |
| **District of Columbia** | 5.9 (5.38 - 6.42) | 2.67 (1.87 - 3.69) |
| **Florida** | 3.78 (3.71 - 3.85) | 2.62 (2.48 - 2.76) |
| **Georgia** | 3.83 (3.72 - 3.94) | 1.88 (1.7 - 2.06) |
| **Hawaii** | 6.78 (6.43 - 7.13) | 5.73 (4.89 - 6.57) |
| **Idaho** | 3.47 (3.22 - 3.72) | 2.69 (2.18 - 3.2) |
| **Illinois** | 4.47 (4.38 - 4.57) | 2.8 (2.6 - 3) |
| **Indiana** | 3.83 (3.7 - 3.95) | 3.54 (3.24 - 3.85) |
| **Iowa** | 3.75 (3.59 - 3.92) | 2.36 (2 - 2.72) |
| **Kansas** | 2.53 (2.38 - 2.68) | 2.34 (1.97 - 2.71) |
| **Kentucky** | 1.47 (1.37 - 1.56) | 1.13 (0.92 - 1.34) |
| **Louisiana** | 3.8 (3.65 - 3.95) | 2.31 (2 - 2.62) |
| **Maine** | 2.73 (2.51 - 2.95) | 1.61 (1.23 - 2.06) |
| **Maryland** | 4.16 (4.02 - 4.3) | 2.66 (2.39 - 2.93) |
| **Massachusetts** | 1.71 (1.63 - 1.79) | 1.36 (1.18 - 1.55) |
| **Michigan** | 5.02 (4.9 - 5.13) | 2.68 (2.46 - 2.9) |
| **Minnesota** | 3.27 (3.14 - 3.39) | 1.98 (1.73 - 2.22) |
| **Mississippi** | 3.15 (2.99 - 3.32) | 1.95 (1.59 - 2.31) |
| **Missouri** | 3.57 (3.45 - 3.7) | 2.37 (2.11 - 2.63) |
| **Montana** | 2.02 (1.8 - 2.24) | 1.77 (1.29 - 2.37) |
| **Nebraska** | 1.94 (1.78 - 2.1) | 2.85 (2.34 - 3.37) |
| **Nevada** | 6.29 (6.04 - 6.55) | 2.99 (2.57 - 3.41) |
| **New Hampshire** | 2.22 (2.01 - 2.42) | 1.2 (0.85 - 1.64) |
| **New Jersey** | 3.54 (3.44 - 3.64) | 1.8 (1.62 - 1.99) |
| **New Mexico** | 2.85 (2.66 - 3.04) | 2.26 (1.82 - 2.7) |
| **New York** | 2.45 (2.39 - 2.5) | 1.6 (1.48 - 1.72) |
| **North Carolina** | 3.32 (3.23 - 3.42) | 2.4 (2.2 - 2.59) |
| **North Dakota** | 3.74 (3.38 - 4.11) | 2.59 (1.86 - 3.51) |
| **Ohio** | 3.86 (3.77 - 3.95) | 2.71 (2.51 - 2.91) |
| **Oklahoma** | 2.57 (2.43 - 2.7) | 2.65 (2.29 - 3) |
| **Oregon** | 2.72 (2.59 - 2.85) | 2.57 (2.25 - 2.89) |
| **Pennsylvania** | 3.21 (3.13 - 3.28) | 3.02 (2.83 - 3.22) |
| **Rhode Island** | 2.24 (2.02 - 2.46) | 1.62 (1.13 - 2.25) |
| **South Carolina** | 4.69 (4.53 - 4.85) | 3.51 (3.15 - 3.86) |
| **South Dakota** | 2.8 (2.52 - 3.08) | 1.86 (1.31 - 2.55) |
| **Tennessee** | 4.53 (4.39 - 4.67) | 3.42 (3.11 - 3.72) |
| **Texas** | 3.45 (3.39 - 3.52) | 2.58 (2.45 - 2.71) |
| **Utah** | 3.1 (2.9 - 3.3) | 3.9 (3.39 - 4.41) |
| **Vermont** | 4.04 (3.65 - 4.42) | 2.41 (1.68 - 3.35) |
| **Virginia** | 2.8 (2.7 - 2.9) | 1.74 (1.55 - 1.92) |
| **Washington** | 5.44 (5.3 - 5.59) | 4.3 (3.99 - 4.62) |
| **West Virginia** | 2.29 (2.12 - 2.46) | 1.62 (1.26 - 2.05) |
| **Wisconsin** | 3.76 (3.63 - 3.89) | 2.56 (2.29 - 2.83) |
| **Wyoming** | 2.24 (1.91 - 2.57) | 2.89 (2 - 4.04) |
