# Supplementary material for: Trends and disparities in dilated cardiomyopathy related mortality among adults in the United States: A CDC WONDER analysis (1999–2023)
Source: PLoS One. 2025 Oct 16;20(10):e0333525. doi: 10.1371/journal.pone.0333525 (PMC12530569; doi:10.1371/journal.pone.0333525)
Supplement: S6 Table — (DOCX) [file pone.0333525.s006.docx]

**Supplemental Table 6: Dilated Cardiomyopathy Related Age-Adjusted Mortality Rates per 100,000, Stratified by Census Region in the United States, 1999 to 2023.**

| **Census Region** | **Year** | **Age-Adjusted Rate (95% CI)** |
| --- | --- | --- |
| **Northeast** |  |  |
| **Northeast** | 1999 | 4.07 (4.27-3.86) |
| **Northeast** | 2000 | 3.81 (4.01-3.61) |
| **Northeast** | 2001 | 3.67 (3.86-3.47) |
| **Northeast** | 2002 | 3.36 (3.54-3.17) |
| **Northeast** | 2003 | 2.98 (3.16-2.81) |
| **Northeast** | 2004 | 4.23 (4.43-4.02) |
| **Northeast** | 2005 | 3.69 (3.88-3.5) |
| **Northeast** | 2006 | 3.53 (3.71-3.34) |
| **Northeast** | 2007 | 3.16 (3.33-2.98) |
| **Northeast** | 2008 | 3.12 (3.3-2.95) |
| **Northeast** | 2009 | 2.94 (3.11-2.78) |
| **Northeast** | 2010 | 2.61 (2.76-2.45) |
| **Northeast** | 2011 | 2.49 (2.64-2.34) |
| **Northeast** | 2012 | 2.2 (2.34-2.06) |
| **Northeast** | 2013 | 2.27 (2.41-2.12) |
| **Northeast** | 2014 | 1.91 (2.05-1.78) |
| **Northeast** | 2015 | 1.91 (2.05-1.78) |
| **Northeast** | 2016 | 1.9 (2.04-1.77) |
| **Northeast** | 2017 | 1.69 (1.81-1.57) |
| **Northeast** | 2018 | 1.61 (1.73-1.49) |
| **Northeast** | 2019 | 1.6 (1.72-1.48) |
| **Northeast** | 2020 | 1.78 (1.9-1.65) |
| **Northeast** | 2021 | 1.82 (1.95-1.7) |
| **Northeast** | 2022 | 2.05 (2.18-1.91) |
| **Northeast** | 2023 | 1.83 (1.96-1.7) |
| **Midwest** |  |  |
| **Midwest** | 1999 | 5.73 (5.96-5.5) |
| **Midwest** | 2000 | 5.14 (5.35-4.92) |
| **Midwest** | 2001 | 4.88 (5.09-4.67) |
| **Midwest** | 2002 | 4.94 (5.15-4.73) |
| **Midwest** | 2003 | 4.39 (4.59-4.2) |
| **Midwest** | 2004 | 5.74 (5.97-5.52) |
| **Midwest** | 2005 | 5.59 (5.81-5.37) |
| **Midwest** | 2006 | 4.86 (5.06-4.65) |
| **Midwest** | 2007 | 4.77 (4.97-4.56) |
| **Midwest** | 2008 | 4.55 (4.75-4.36) |
| **Midwest** | 2009 | 4.16 (4.35-3.98) |
| **Midwest** | 2010 | 3.91 (4.09-3.73) |
| **Midwest** | 2011 | 3.53 (3.7-3.36) |
| **Midwest** | 2012 | 3.43 (3.59-3.26) |
| **Midwest** | 2013 | 3.27 (3.43-3.1) |
| **Midwest** | 2014 | 2.88 (3.03-2.72) |
| **Midwest** | 2015 | 2.77 (2.91-2.62) |
| **Midwest** | 2016 | 2.83 (2.98-2.68) |
| **Midwest** | 2017 | 2.84 (2.99-2.69) |
| **Midwest** | 2018 | 2.77 (2.91-2.62) |
| **Midwest** | 2019 | 2.48 (2.61-2.34) |
| **Midwest** | 2020 | 2.95 (3.1-2.8) |
| **Midwest** | 2021 | 2.88 (3.03-2.73) |
| **Midwest** | 2022 | 2.68 (2.82-2.53) |
| **Midwest** | 2023 | 2.42 (2.55-2.28) |
| **South** |  |  |
| **South** | 1999 | 5.27 (5.45-5.09) |
| **South** | 2000 | 5.21 (5.39-5.03) |
| **South** | 2001 | 4.88 (5.05-4.71) |
| **South** | 2002 | 4.48 (4.65-4.32) |
| **South** | 2003 | 4.22 (4.37-4.06) |
| **South** | 2004 | 5.66 (5.84-5.48) |
| **South** | 2005 | 5.31 (5.48-5.14) |
| **South** | 2006 | 4.66 (4.82-4.5) |
| **South** | 2007 | 4.23 (4.38-4.08) |
| **South** | 2008 | 3.99 (4.13-3.84) |
| **South** | 2009 | 3.71 (3.85-3.58) |
| **South** | 2010 | 3.44 (3.57-3.31) |
| **South** | 2011 | 3.0 (3.12-2.88) |
| **South** | 2012 | 2.67 (2.79-2.56) |
| **South** | 2013 | 2.52 (2.63-2.41) |
| **South** | 2014 | 2.41 (2.51-2.3) |
| **South** | 2015 | 2.42 (2.53-2.31) |
| **South** | 2016 | 2.31 (2.41-2.21) |
| **South** | 2017 | 2.28 (2.38-2.18) |
| **South** | 2018 | 2.22 (2.32-2.12) |
| **South** | 2019 | 2.3 (2.4-2.2) |
| **South** | 2020 | 2.39 (2.49-2.29) |
| **South** | 2021 | 2.52 (2.62-2.41) |
| **South** | 2022 | 2.32 (2.42-2.23) |
| **South** | 2023 | 2.31 (2.41-2.22) |
| **West** |  |  |
| **West** | 1999 | 5.49 (5.73-5.25) |
| **West** | 2000 | 4.88 (5.11-4.66) |
| **West** | 2001 | 4.52 (4.74-4.31) |
| **West** | 2002 | 4.58 (4.79-4.37) |
| **West** | 2003 | 4.58 (4.79-4.37) |
| **West** | 2004 | 5.19 (5.41-4.97) |
| **West** | 2005 | 5.02 (5.24-4.8) |
| **West** | 2006 | 4.68 (4.88-4.47) |
| **West** | 2007 | 4.3 (4.49-4.1) |
| **West** | 2008 | 4.32 (4.51-4.12) |
| **West** | 2009 | 4.06 (4.25-3.87) |
| **West** | 2010 | 3.91 (4.09-3.73) |
| **West** | 2011 | 3.86 (4.04-3.69) |
| **West** | 2012 | 3.8 (3.98-3.63) |
| **West** | 2013 | 3.63 (3.8-3.47) |
| **West** | 2014 | 3.48 (3.64-3.31) |
| **West** | 2015 | 3.4 (3.56-3.24) |
| **West** | 2016 | 3.18 (3.34-3.03) |
| **West** | 2017 | 3.31 (3.46-3.15) |
| **West** | 2018 | 2.87 (3.01-2.73) |
| **West** | 2019 | 2.89 (3.04-2.75) |
| **West** | 2020 | 3.15 (3.3-3.0) |
| **West** | 2021 | 3.38 (3.54-3.22) |
| **West** | 2022 | 3.19 (3.34-3.04) |
| **West** | 2023 | 2.69 (2.82-2.55) |
