# Supplementary material for: Trends and disparities in dilated cardiomyopathy related mortality among adults in the United States: A CDC WONDER analysis (1999–2023)
Source: PLoS One. 2025 Oct 16;20(10):e0333525. doi: 10.1371/journal.pone.0333525 (PMC12530569; doi:10.1371/journal.pone.0333525)
Supplement: S7 Table — (DOCX) [file pone.0333525.s007.docx]

**Supplemental Table 7: Dilated Cardiomyopathy Related Age-Adjusted Mortality Rates per 100,000 in United States stratified by Urban-Rural Classification, 1999-2020**

|  | **Age-Adjusted Rate (95% CI)** | |
| --- | --- | --- |
| **Year** | **Urban** | **Rural** |
| **1999** | 5.31 (5.2 - 5.43) | 4.55 (4.32 - 4.78) |
| **2000** | 4.94 (4.82 - 5.05) | 4.38 (4.15 - 4.61) |
| **2001** | 4.63 (4.52 - 4.74) | 4.15 (3.93 - 4.37) |
| **2002** | 4.41 (4.31 - 4.52) | 4.09 (3.87 - 4.31) |
| **2003** | 4.12 (4.02 - 4.23) | 3.77 (3.56 - 3.97) |
| **2004** | 5.3 (5.18 - 5.41) | 5.19 (4.94 - 5.43) |
| **2005** | 4.96 (4.86 - 5.07) | 4.9 (4.67 - 5.14) |
| **2006** | 4.56 (4.45 - 4.66) | 4.12 (3.9 - 4.33) |
| **2007** | 4.13 (4.03 - 4.23) | 4.17 (3.95 - 4.38) |
| **2008** | 3.98 (3.88 - 4.07) | 4.11 (3.9 - 4.32) |
| **2009** | 3.73 (3.63 - 3.82) | 3.7 (3.5 - 3.91) |
| **2010** | 3.45 (3.36 - 3.53) | 3.58 (3.38 - 3.78) |
| **2011** | 3.22 (3.13 - 3.3) | 3.24 (3.05 - 3.43) |
| **2012** | 2.99 (2.91 - 3.07) | 2.97 (2.79 - 3.15) |
| **2013** | 2.89 (2.81 - 2.96) | 2.88 (2.7 - 3.06) |
| **2014** | 2.64 (2.57 - 2.72) | 2.85 (2.67 - 3.03) |
| **2015** | 2.58 (2.51 - 2.65) | 2.91 (2.73 - 3.09) |
| **2016** | 2.51 (2.43 - 2.58) | 2.81 (2.63 - 2.99) |
| **2017** | 2.52 (2.45 - 2.59) | 2.66 (2.49 - 2.83) |
| **2018** | 2.34 (2.27 - 2.4) | 2.65 (2.48 - 2.81) |
| **2019** | 2.36 (2.29 - 2.42) | 2.41 (2.25 - 2.58) |
| **2020** | 2.53 (2.46 - 2.6) | 2.89 (2.71 - 3.06) |
